# Supplementary material for: Nationwide screening for Fabry disease in unselected stroke patients
Source: PLoS One. 2021 Dec 14;16(12):e0260601. doi: 10.1371/journal.pone.0260601 (PMC8670679; doi:10.1371/journal.pone.0260601)
Supplement: Attachment — Submitted filename: Response to Reviewers.docx [file pone.0260601.s001.docx]

Tomohiko Ai, M.D., Ph.D.
Academic Editor
PLOS ONE

Response to the reviewers

PONE-D-21-10014
Nationwide screening for Fabry disease in unselected stroke patients
PLOS ONE

Dear professor Ai,

We thank You and Your journal for your very helpful and constructive comments. We have tried our best to answer them and mainly to correct our article accordingly.

With best regards,

Ales Tomek

**Reviewer #1 comments and our answers:** 
• The authors should specify that the discriminant is not the age per se, but the age of onset of the stroke event/s. So, it would read better if they would specify that FD screening is appropriate for all subjects that had a stroke event before 50 years of age

- Corrected according to the reviewer. We agree that it is more valid and clear.

• Given the limitation of the algorithm employing enzymatic activity and globotriaosylsphingosine (lyso-Gb3) quantification before genetic analysis in males, should they consider suggesting doing genetic testing for GLA in all subjects that had a stroke event before 50 years of age irrespective of biochemical analysis?

- We agree that the genetic analysis it is the most valid method of screening in both, females and males. We did not apply the genetic testing as the first line method in males only because of the financial reasons. We are aware of the shortcomings of our approach, and we mentioned it in the discussion. Although, this approach was used by many previous studies - e.g., Kinoshita et al 2018, Romani et al 2015 and also is in agreement with a European expert recommendation (Gal et al. J Inherit Metab Dis. 2011; 34(2): 509–514). We added a clarifying sentence in the discussion according to the very valid reviewer ‘s remark.

• In the Methods section, the authors stated that they used: “The GLA gene was analyzed by PCR and sequencing (NGS-Illumina) of the entire coding region and the highly conserved exon-intron splice junctions”. Could they provide more details about the platform and technical specifics used (i.e.: MiSeq, HiSeq, depth of coverage, bioinformatic analysis, etc.) and if copy number variants (CNV) have been analyzed. CNV are rare but can be identified in up to 5% of cases (see: <https://www.ncbi.nlm.nih.gov/books/NBK1292/#fabry.Summary>). If CNV was not done, please add to the limitations.

- We thank to the reviewer for a very valid remark. The *GLA* gene was analyzed by an amplicon-based next-generation sequencing approach. The amplicons cover the entire coding region and the highly conserved exon-intron splice junctions. The libraries are  sequenced on an Illumina platform (MiSeq).  An in-house bioinformatics pipeline including read alignment to GRCh37/hg19 genome assembly, variant calling and annotation is used. We have a minimum coverage of >20x for every amplicon. Missing regions or regions of poor quality are completed with classical Sanger sequencing to achieve 100% coverage. Variants of relevance identified by NGS are continuously and individually in-house validated for quality aspects; those variants which meet our internal QC criteria (based on extensive validation processes) are not validated by Sanger. CNV analysis was not performed on this NGS data. When deletions or duplications were suspected, this was done by MLPA (MRC Holland kit. but it was not a standard procedure in this project.
- We added the requested details and limitations.

• While the GLA variant c.1235_1236del (p.Thr412Serfs*) is an established pathogenic change (<https://www.ncbi.nlm.nih.gov/clinvar/variation/198402/>), for the c.973G>A (p.Gly325Ser) (aka G325S) in ClinVar there is conflicting classification (<https://www.ncbi.nlm.nih.gov/clinvar/variation/92573/>), If considered Pathogenic or Likely Pathogenic, please provide your classification and rationale using the ACMG 2015 guidelines (PMID: 25741868) and any professional updated guidelines.

- c.973G>A (p.Gly325Ser) is considered by International Fabry Disease Genotype-Phenotype Database (dbFGP) at <http://www.dbfgp.org/> as  type 2 Later-Onset mutation that encodes an α-Gal A enzyme with a small amount of residual activity in males. Affected males present with a later-onset phenotype and require the enzyme replacement treatment.
- c.973G>A replaces glycine with serine at codon 325 of the GLA protein (p.Gly325Ser). The glycine residue is highly conserved and there is a physicochemical difference between glycine and serine. This variant is not present in population databases (ExAC no frequency). This variant has been observed in individual with Fabry disease (Lukas et al 2013, PMID: 23935525). ClinVar contains an entry for this variant (Variation ID: 92573). Experimental studies have shown that this variant affects GLA protein function (Lukas et al 2013, PMID: 23935525). This variant disrupts the p.Gly325 amino acid residue in GLA. Other variant(s) that disrupt this residue have been determined to be pathogenic (Ries et al 2005 PMID: 15713906, Schäfer et al 2005 PMID: 15776423, and Wu et al 2011, PMID: 21598360).
- Our opinion is that c.973G>A variant should be classified according to the ACMG 2015 guidelines based on the above facts as **pathogenic, as it has two** **strong criteria – PS1 and PS3**. Besides those two strong criteria, there is 1 moderate (PM2), and two supporting criteria (PP4, PP5).
- Based on this data and also on the basis of Fabry phenotype in our patient, we decided to consider the variant pathogenic

• Although still erroneously use in official publication and mistakenly tolerated by scientific journals, the official term is variant, not mutation (see: PMID: 25741868 under “Terminology”)

- We thank for the comment. Corrected as appropriate.

• The authors should specify that the c.937G>T (p.Asp313Tyr) (aka D313Y) is a common pseduodeficiency allele that does not cause disease, but individuals with this variant can exhibit low alpha-galactosidase activity during enzyme analysis. As far as the other variants, the authors correctly identified them as potentially benign or variants of uncertain significance (VUS). In the case of the two novel GLA variants, p.Arg30Lys and p.Arg38Gly, although the age at testing (please provide the age at stroke event onset, if known) is favoring a more tolerated role, the authors should discuss the fact that, most females (not only with regard to those two variants, but in general), harboring a GLA variant, may or may not present the whole collection of symptoms, due to absolute gene dosage (heterozygous versus hemizygous in males) and the phenomenon of X-inactivation

- We agree with the reviewer that c.937G>T is a common allele without any known association with a classical Fabry phenotype. We have stated in the discussion our reasoning that it might represent a low-effect stroke risk factor. If it is contributing to the overall stroke risk in its carriers, or it is an accidental finding, remains to be fully explained by further large populational studies.
- As the study was prospective and carried in the stroke units, the age at testing is the same as the age at stroke event.

• Was all the participant ancestry background uniform, or were there a significant ancestry background admixture? Some variants may be common in population isolates due to founder effect

- We are not aware of any significant background ancestry admixture. Although, we did not collect the ancestry or nationality/origin data. All screened patients were Czech citizens. In general, the Czech Republic population is considered fairly uniform – Central or Eastern European of mixed Slavic, Celtic and Germanic origin.

• Please use the most current human genome variation nomenclature (<https://varnomen.hgvs.org/>)

- Corrected accordingly.
